# Pathogenic BCL11A variants provide insights into the mechanisms of human fetal hemoglobin silencing

**DOI:** 10.1371/journal.pgen.1009835

**Published:** 2021-10-11

**Authors:** Yong Shen, Rick Li, Kristian Teichert, Kara E. Montbleau, Jeffrey M. Verboon, Richard A. Voit, Vijay G. Sankaran

**Affiliations:** 1 Division of Hematology/Oncology, Boston Children’s Hospital and Department of Pediatric Oncology, Dana-Farber Cancer Institute, Harvard Medical School, Boston, Massachusetts, United States of America; 2 Broad Institute of MIT and Harvard, Cambridge, Massachusetts, United States of America; 3 Harvard Stem Cell Institute, Cambridge, Massachusetts, United States of America; Pennsylvania State University, UNITED STATES

## Abstract

Increased production of fetal hemoglobin (HbF) can ameliorate the severity of sickle cell disease and β-thalassemia. BCL11A has been identified as a key regulator of HbF silencing, although its precise mechanisms of action remain incompletely understood. Recent studies have identified pathogenic mutations that cause heterozygous loss-of-function of BCL11A and result in a distinct neurodevelopmental disorder that is characterized by persistent HbF expression. While the majority of cases have deletions or null mutations causing haploinsufficiency of BCL11A, several missense variants have also been identified. Here, we perform functional studies on these variants to uncover specific liabilities for BCL11A’s function in HbF silencing. We find several mutations in an N-terminal C2HC zinc finger that increase proteasomal degradation of BCL11A. We also identify a distinct C-terminal missense variant in the fifth zinc finger domain that we demonstrate causes loss-of-function through disruption of DNA binding. Our analysis of missense variants causing loss-of-function *in vivo* illuminates mechanisms by which BCL11A silences HbF and also suggests potential therapeutic avenues for HbF induction to treat sickle cell disease and β-thalassemia.

## Author summary

Greater production of fetal hemoglobin can ameliorate the severity of the most common genetic disorders that impact the hemoglobin genes, sickle cell disease and β-thalassemia. BCL11A has been shown to act as a critical factor that silences fetal hemoglobin in adult cells. Clinical trials are currently seeking to target BCL11A for induction of fetal hemoglobin to treat individuals impacted by sickle cell disease and β-thalassemia. How best to do this remains unclear. Rare haploinsufficient mutations in BCL11A cause a neurodevelopmental disorder with elevated fetal hemoglobin levels. Here, we have examined the functional consequences of pathogenic missense mutations in BCL11A and gained insight into how these mutations can alter the function of this key factor. We find that many mutations increase degradation of the BCL11A protein by the proteasome. One mutation specifically impairs DNA binding. These findings provide potential insights into strategies that could be employed to induce fetal hemoglobin for therapeutic purposes.

## Introduction

Functional follow up on the results of genome-wide association studies examining human variation in fetal hemoglobin (HbF) expression has led to the identification of BCL11A as a key HbF silencing factor and regulator of the developmental fetal-to-adult hemoglobin switch [1–6]. Recent studies have provided additional details on the mechanisms by which BCL11A acts to silence HbF. These studies have identified co-factors that cooperate with BCL11A [4,7], DNA binding sites for BCL11A within the HbF-encoding *HBG1/2* (γ-globin) promoters [8,9], and potential long-range interactions mediated by BCL11A [10,11]. Recent functional analyses through combinatorial genome editing in single erythroid progenitor cells have revealed how BCL11A acts at both the *HBG1/2* promoter elements and through distal interactions to effectively silence HbF [12]. In addition, transcriptional regulatory elements that are important for erythroid-specific expression of BCL11A [13,14] and upstream developmental regulators of *BCL11A* mRNA translation have been identified [15]. Despite the substantial progress, the precise mechanisms by which BCL11A silences *HBG1/2* expression remain to be fully defined.

The critical role of BCL11A in HbF silencing has been validated *in vivo* through the identification of patients with pathogenic loss-of-function mutations [16–20]. All of these mutations were identified as *de novo* events in individuals who were diagnosed with distinct neurodevelopmental phenotypes [21,22]. When measured, individuals with pathogenic variants in BCL11A have demonstrated consistently elevated HbF levels. Deletions causing these neurodevelopmental phenotypes result in haploinsufficiency for BCL11A, which thereby results in elevated HbF levels in the context of the hematopoietic system [16,17]. In addition to the deletions and a number of definite loss-of-function mutations, four likely pathogenic missense variants in BCL11A have also been reported to date [18,19,21]. We reasoned that the functional study of these variants could provide additional insights into the mechanisms underlying HbF silencing by BCL11A and help define pathogenic mechanisms. Moreover, detailed study of the mechanisms through which these missense variants act may help identify opportunities to interfere with BCL11A activity for therapeutic HbF induction.

## Materials and methods

### Isolation of cord blood CD34+ hematopoietic stem and progenitor cells (HSPCs)

CD34^+^ HSPCs from human cord blood were isolated by positive magnetic selection using the EasySep Human CD34 Positive Selection Kit II (Stem Cell Technologies) after mononuclear cell isolation on a Ficoll-Paque density gradient (Stem Cell Technologies). The purity of isolated cells was assessed by flow cytometry with a conjugated anti-human CD34 antibody. Freshly isolated CD34^+^ HSPCs were either immediately cultured or cryopreserved for later use.

### Erythroid differentiation

CD34^+^ HSPCs were cultured and differentiated in a three-phase erythroid differentiation culture system, as previously described[15,23,24]. Briefly, cells were cultured in Iscove’s modified Dulbecco’s medium (IMDM, Life Technologies) containing 200 ug/mL human holo-transferrin, 10 ug/mL recombinant human insulin, 3 IU/mL heparin, 2% human AB plasma, 3% human AB serum and 1% penicillin/streptomycin (base medium). In phase I (0–7 d), the culture were supplemented with 3 IU/mL EPO, 10 ng/mL SCF, 1 ng/mL IL-3 and in phase II (7–12 d) they were supplemented with 3 IU/mL EPO and 10 ng/mL SCF alone. In phase III (12–17 d), primary cell cultures contained 1 mg/ml of human holo-transferrin supplemented with 3 IU/ml EPO. Cells were maintained at 10^5^−10^6^ per ml in phases I and II, and at 1–5 x 10^6^ per ml in phase III. Cells were changed into fresh culture medium every 3 days. For assessment of differentiation, cells were washed in PBS and stained with anti-human CD49d, CD71 and CD235a antibodies. Flow cytometric analyses were conducted on an Accuri C6 instrument and all data were analyzed using FlowJo software (v.10.3). S2 Table lists the antibodies used for flow cytometry.

### Creation of BCL11A mutant constructs

All constructs incorporating the single nucleotide variants of interest were created using the Q5 Site-Directed Mutagenesis Kit (New England Biolabs) following the manufacturer’s recommendations. HA-tags were introduced to the C-terminus of the BCL11A WT and Mut-4 constructs via Q5 Site-Directed Mutagenesis Kit (New England Biolabs) following the manufacturers recommendations for insertions. A glycine-serine linker (G-S-S-G) was added at the C-terminal end of the HA-tag. Primers used for constructs are listed in S1 Table.

### Lentiviral infections

HSPCs undergoing erythroid differentiation were transduced with the HMD empty vector control, BCL11A WT and Mutant 1–4 lentiviral constructs on day 2 of differentiation; 293T cells for lentivirus production were cultured in DMEM (Life Technologies) with 10% FBS and 1% penicillin/streptomycin. Approximately 24 hours before transfection, 293T cells were seeded, without antibiotics, in six-well plates. Cells were co-transfected with the packaging vectors pVSVG, pΔ8.9, and the lentiviral genomic vector of interest. The medium was changed to base medium on the day following transfection, and the viral supernatant was collected approximately 48 hours post-transfection, filtered with a 0.45-μm filter, and used for infection of CD34^+^ cells. Where the virus was required to be concentrated, 293T cells were seeded in 10-cm dishes and the viral supernatant was filtered and centrifuged at 24,000 r.p.m. for 2 h at 4°C. Between 200,000 and 300,000 CD34^+^ cells or K562 cells were infected in six-well plates with 8 μg/ml of polybrene (Millipore), spun at 2,000 r.p.m. for 1.5 h at room temperature, and incubated in the viral supernatant overnight at 37°C. Virus was washed off 1 day after infection, and infected cells were selected for by GFP expression driven by IRES-GFP in the HMD vector in the particular construct. GFP^+^ cells were sorted by fluorescence-activated cell sorting (FACS) in a sterile manner and cultured for further analysis. Flow cytometric analyses were conducted on Becton Dickinson LSRII and data were analyzed using FlowJo software (v.10.3).

### Cycloheximide chase experiments

K562 cells transduced with the HMD empty vector control, BCL11A WT, and Mutant 1–4 lentiviral constructs were then treated with cycloheximide (Sigma Aldrich) at 25 μg/ml to inhibit protein synthesis, and harvested at the indicated time points following treatment. BCL11A protein was analyzed by immunoblotting, and the intensities of BCL11A protein bands were quantified using ImageJ. GAPDH was used as a control.

### Proteasome Inhibitor MG-132 treatment

K562 cells transduced with the HMD empty vector control, BCL11A WT, and Mutant 1–4 lentiviral constructs were then treated with MG-132 (Millipore) at 10 uM to inhibit proteasome-mediated degradation of protein, and harvested at the 4hr time point following treatment. Adult blood HSPCs at day 8 of the differentiation process were treated with MG-132 (Millipore) at 5 uM to inhibit the proteasome, and harvested at the indicated time points following treatment. BCL11A protein was analyzed by immunoblotting, and the intensities of BCL11A protein bands were quantified using ImageJ. GAPDH was used as a control.

### Cellular Thermal Shift Assay (CETSA)

Thirty million K562 cells transduced with HMD control, BCL11A WT, and Mutant 1–4 lentiviral constructs were harvested by centrifugation at 300 r.c.f. for 10 minutes and 4°C, and washed twice with PBS, pH 7.4. The cells were resuspended at 30 x 10^6^ cells/mL in PBS containing PMSF. Each cell line was aliquoted into PCR tubes at 100 μL. Individual aliquots of cells were heated to 27°C, 37°C, 47°C, 57°C, and 67°C for 3 minutes. Cells were then cooled at room temperature for 3 minutes, and frozen at -80°C. Lysates were prepared by 3 freeze-thaw cycles, followed by vortexing. Cellular debris and protein aggregates were pelleted, and 90 μL supernatant was collected in fresh tubes for immunoblot analysis.

### Immunoblot analysis

Cells were lysed in RIPA buffer (Santa Cruz Biotechnology) and protein levels were quantified with the DC Protein Assay (BioRad). Briefly, samples were incubated at 95°C for 5 min in 4x Laemmli sample buffer (BioRad) and loaded onto a Mini-Protein TGX Gel (BioRad) for electrophoresis at 120V for 1 hr. Proteins were then transferred to PVDF membrane with BioRad wet transfer system at 100V for 1.5 hr. Membranes were blocked with TBS-T/3% BSA for 1 hr and then incubated with primary antibodies overnight in a cold room with shaking. Excess antibodies were washed with TBS-T (50 mM Tris pH 8.0, 150 mM NaCl, 1% Tween 20) 3 times and HRP-conjugated secondary antibodies were incubated for 30 min at room temperature. After 3 washes with TBS-T, the membranes were developed with Clarity Western ECL Substrate kit (BioRad). Immunoblots were performed with antibodies targeting BCL11A (14B5, no. ab19487, AbCam, at 1/1,000 dilution), anti-HA tag (no. ab9110, AbCam, at 1/1,000 dilution) and GAPDH (6C5, no. sc-32233, Santa Cruz, at 1/5,000 dilution). The secondary antibodies used were goat anti-rabbit (BioRad) and goat anti-mouse (BioRad). S2 Table lists the antibodies used for Immunoblots.

### RNA isolation and quantitative PCR with reverse transcription (RT-qPCR)

RNA was isolated using the RNeasy Mini Kit (Qiagen) with on-column DNAse (Qiagen) digestion, according to the manufacturer’s instructions. cDNA was synthesized with the iScript cDNA synthesis kit (BioRad) in a total volume of 20 μl according to the manufacturer’s instructions. RT-qPCR was carried out using a 96-well plate on a CFX96 Real Time System (BioRad) with iQ SYBR Green Supermix (BioRad). Gene-specific primers used for RT-qPCR are listed in S1 Table.

### HbF flow cytometry

For HbF analysis cells were fixed in 0.05% glutaraldehyde for 10 min, washed 2 times with PBS/0.1%BSA (Sigma), and permeabilized with 0.1% Triton X-100 (Life Technologies, prepared in PBS/0.1%BSA) for 5 minutes. Following one wash with PBS/0.1% BSA, cells were stained with HbF-APC conjugate antibody (Invitrogen). For primary erythroid cells, 5 × 10^5^ cells were incubated with 0.4 μg HbF-APC antibody for 10 minutes in the dark at room temperature. Cells were then washed twice with PBS/0.1%BSA. Flow cytometric analyses were conducted on an Accuri C6 instrument and all data were analyzed using FlowJo software (v.10.3).

### Hemoglobin HPLC

Approximately 5 x 10^6^ erythroblasts were collected on day 17 of HSPC erythroid differentiation and subjected to lysis to free hemoglobin for hemoglobin high performance liquid chromatography (HPLC). HbF levels were measured using a G7 HPLC Analyzer (Tosoh Bioscience, Inc.) with the beta-thalassemia program. To compare HbF levels across experiments, we defined HbF% as a proportion of HbF relative to HbA and other hemoglobin subtypes.

### Structural modeling

The sequence for the N-terminal C2HC domain, amino acids 771–793, was submitted to I-TASSER to generate structural models for this region, which was structurally predicted as a zinc finger. A published structure for the C-terminal zinc fingers 4–6 was used to generate a model for Mutant 4 (PDB: 6KI6). Briefly, the mutagenesis tool in PyMOL was used to model the mutant p.Lys784Thr, substituting a threonine residue for lysine at position 784. The structural models were visualized in, and all images were generated using PyMOL.

### Subcellular localization studies

Cord blood CD34^+^ HSPCs were cultured until day 11 of differentiation. Nuclear and cytoplasmic fractionation was performed with the PARIS kit (no. AM1921, Ambion) according to the manufacturer’s instructions. Localization immunoblots were visualized using the LI-COR Odyssey DLx system. Immunoblots were performed with primary antibodies anti-HA tag (no. ab9110, AbCam, at 1/1,000 dilution), LMNB1 (no. sc-374015, Santa Cruz, at 1/1,000 dilution), and GAPDH (6C5, no. sc-32233, Santa Cruz, at 1/5,000 dilution). Immunoblots used secondary antibodies IRDye 680RD Donkey anti-Mouse IgG Secondary Antibody (926–68072, LI-COR, at 1/5,000 dilution), and IRDye 680RD Donkey anti-Rabbit IgG Secondary Antibody (926–68073, LI-COR, at 1/5,000 dilution) for the appropriate primaries.

### ChIP

Approximately 5 × 10^6^ cells were used for each immunoprecipitation. Cells were prepared with truChIP Chromatin Shearing Kit with Formaldehyde (Covaris) and sonicated with Covaris E220 ultrasonicator (Covaris). Cells were cross-linked with 1% formaldehyde for 2.5 min at room temperature, and the reaction was quenched with glycine at a final concentration of 125 mM. Cross-linked cells were then lysed and sonicated to obtain ~ 200–300 bp fragments of chromatin. Chromatin immunoprecipitations were performed using ChIP-IT High Sensitivity kit (Active Motif). Cross-linked DNA was pulled down at 4°C overnight using anti-HA tag antibody (no. ab9110, AbCam) and control rabbit IgG (no. sc-2027, Santa Cruz). Chromatin cross-linking was then reversed, and DNA was eluted at 65°C overnight and purified. Real-time qPCR was performed on ChIP material on a CFX96 Real Time System (BioRad) with iQ SYBR Green Supermix (BioRad). S1 Table lists the real-time PCR primers used for ChIP-qPCR.

### Statistical analysis

All statistical significance between control and test groups were calculated with two-tailed Student’s t-test. At least three biological replicates are performed. Error bars show SEM. ***P < 0.001 and n.s.: statistically non-significant. All statistical analyses were carried out in GraphPad Prism.

## Results

### Characterization of pathogenic missense variants in BCL11A and their impact on HbF

**Fig 1:**
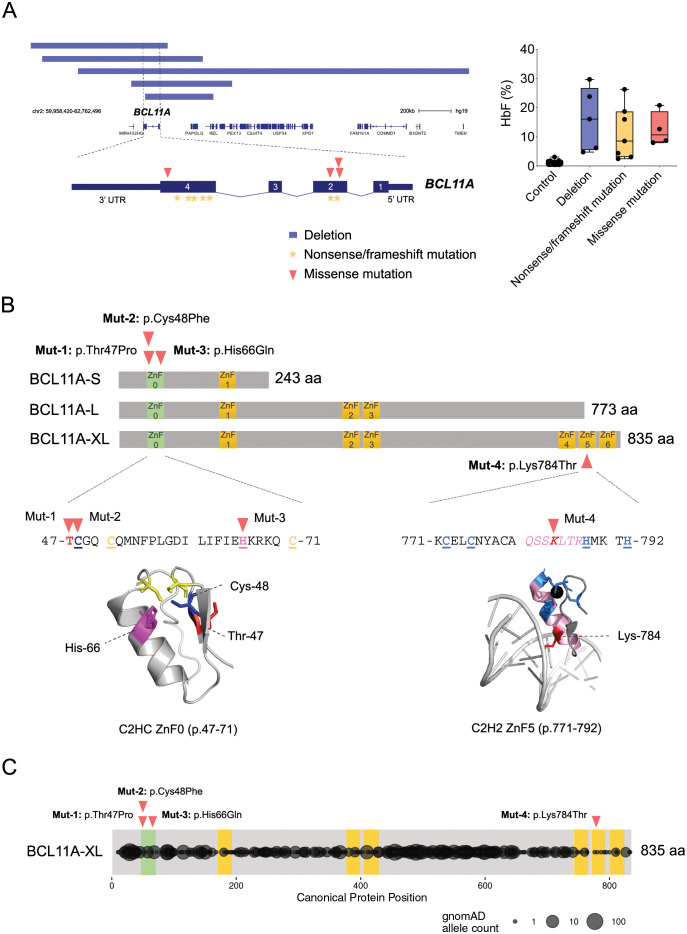
Characterization of pathogenic missense variants in BCL11A. (A) Left panel: Schematic of pathogenic *de novo* deletion, nonsense/frameshift, and missense mutations impacting *BCL11A*. Right panel: Fetal hemoglobin levels of the three categories of variants compared with control cohort. (B) Schematic representation of 4 reported *BCL11A* missense mutations and their locations in a C2HC zinc-finger (ZnF0) at the N-terminal region of BCL11A isoforms (BCL11A-S, BCL11A-L, BCL11A-XL) and the fifth zinc finger domain (ZnF5) at the C-terminal region of BCL11A-XL. (C) Distribution of BCL11A genetic variants from the gnomAD database.

A number of pathogenic *de novo* deletion, loss-of-function, and missense variants impacting *BCL11A* have been reported in individuals diagnosed with a distinct neurodevelopmental disorder that also displays persistence of HbF [16–22,25]. Of these, we identified 16 variants in individuals who have also had HbF levels measured (**Fig 1A and S3 Table**). Importantly, the level of HbF observed in all of these individuals is comparable between those with deletions or null mutations and those who have the missense variants suggesting consistent loss-of-function across all mutation groups (**Fig 1A**). Nevertheless, a range of HbF levels was observed between patients, suggesting some potential variation in the extent to which HbF silencing was perturbed by these mutations. We specifically wondered whether the missense mutations may perturb select functions of BCL11A (e.g. co-factor interactions, DNA binding, or maintenance of protein stability) and could thereby provide important insights into how BCL11A acts to silence HbF in humans *in vivo*.

We initially examined the regions impacted by these mutations. Mut 1–3 (p.Thr47Pro, p. Cys48Phe, and p.His66Gln, respectively) were all located in a predicted C2HC zinc-finger at the N-terminal region of BCL11A (**Fig 1B**). These mutations would impact all of the major isoforms of BCL11A and are all associated with elevated HbF levels. C2HC zinc fingers are not as well characterized as other classes of zinc fingers, but can mediate protein-protein or protein-nucleic acid interactions [26]. Interestingly, this C2HC zinc finger (ZnF0) has been suggested to be dispensable for HbF silencing by the XL isoform of BCL11A in mouse erythroleukemia cells [8]. In addition, introduction of these mutations into the L isoform of BCL11A prevented nuclear localization in HEK293T cells [18]. Of note, the region containing these mutations was predicted to be highly structured based on pairwise free energy calculations [27] and contained minimal variation in the gnomAD database, which is depleted of mutations associated with severe childhood diseases [28] (**Fig 1C**). In addition, we identified Mut 4 (p.Lys784Thr) in the fifth zinc finger domain (ZnF5), which would only impact the XL isoform of BCL11A. ZnF5 has been shown to be critical for DNA binding and a crystal structure that includes this region of the protein has been reported [8,29](**Fig 1B**). Akin to the findings from Mut 1–3, this region was also predicted to be highly structured and depleted of variation in gnomAD (**Fig 1C**).

We would note that while *BCL11A* is depleted of missense and loss-of-function variation in gnomAD (missense Z-score = 3.84, pLI = 0.97) [28], there may be some hypomorphic missense variants found in the population in the absence of neurodevelopmental phenotypes. Unfortunately, since HbF levels are not routinely measured in large population based studies, such as the UK Biobank [30,31], we are unable to readily assess to what extent these variants may result in increased HbF levels. This will be an interesting area for future investigation, but here we have chosen to focus on variants found in individuals with neurodevelopmental phenotypes that are also associated with elevated HbF levels.

### Functional assessment of missense variants impact on HbF silencing

**Fig 2:**
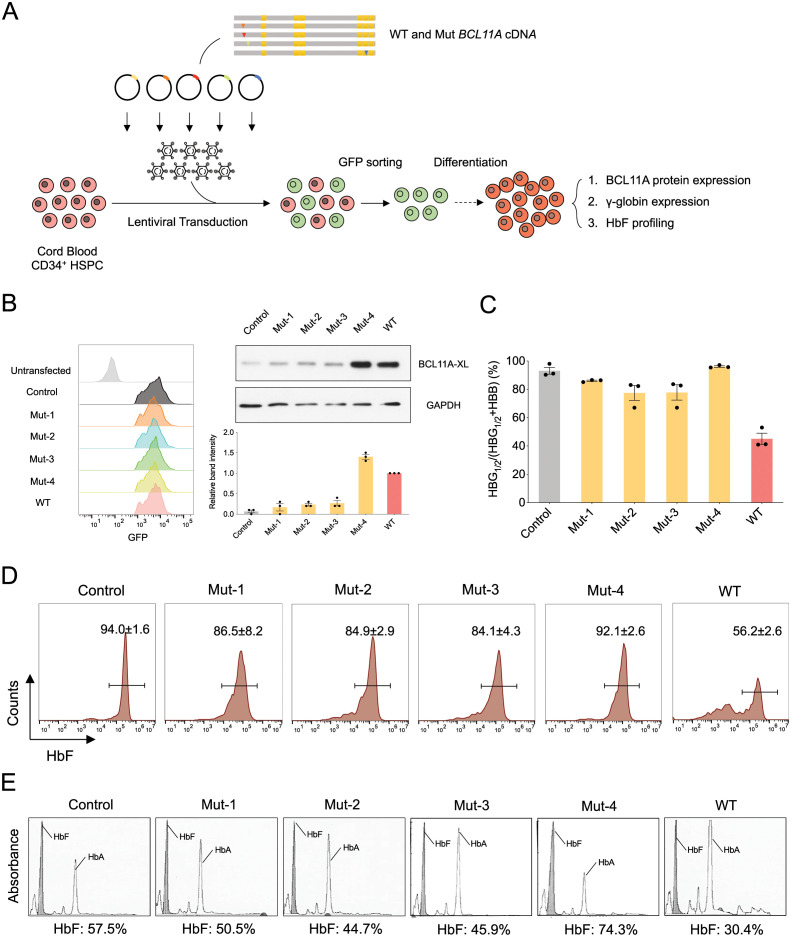
Functional assessment of missense variants impact on HbF silencing. (A) Workflow of exogenous expression of either the wild type or mutant forms of the BCL11A XL in cord blood HSPCs showing lentiviral transduction, GFP sorting, erythroid differentiation and functional analysis. (B) Left panel: A histogram plot shows GFP levels indicating robust and equivalent expression (GFP linked to exogenous BCL11A-XL cDNA). Right panel: A representative western blot and the normalized quantification of BCL11A levels from exogenous cDNA expression in cord blood erythroid cells. BCL11A relative expression levels are normalized with GAPDH expression. Band intensities of all groups are relative to BCL11A-XL WT group. Results are shown as mean ± SEM from three biological replicates. (C) Gene expression analysis for HBG1/2 and HBB in cord blood erythroid cells expressing exogenous isoforms of BCL11A cDNA. Results are shown as mean ± SEM from three biological replicates. (D) Representative HbF flow cytometry on day 13 of cord blood erythroid differentiation. Results are shown as mean ± SEM from three biological replicates. (E) Representative HbF HPLC on day 17 of cord blood erythroid differentiation. HbF: hemoglobin F (fetal form); HbA: hemoglobin A (adult form). HbF%: HbF as a fraction of HbA plus HbF.

We have shown that physiologic expression of the XL isoform of BCL11A can effectively silence *HBG1/2* expression in erythroid cells derived from primary cord blood CD34^+^ hematopoietic stem and progenitor cells (HSPCs) [12,15]. We therefore utilized lentiviral transduction of cord blood HSPCs with either the wild type or mutant forms of the *BCL11A* XL cDNA and promoted semi-synchronous erythroid differentiation using a well-established *in vitro* culture approach [23,32,33] (**Fig 2A**). We sorted cells that were transduced with a co-transcribed GFP marker, which was encoded following an internal ribosome entry site after the *BCL11A* cDNA (**S1A and S1B Fig**). We noted no difference in erythroid differentiation using phenotypic surface markers, including CD235a and CD71, at day 10 of the differentiation process (**S1C Fig**). Interestingly, when we assessed protein expression of BCL11A in the transduced cells, we noted low expression of Muts 1–3, despite robust expression of GFP encoded within the same mRNA (**Figs 2B and** S1B). In contrast, Mut 4 had a distinct pattern and was well expressed to a greater extent than the wild type BCL11A XL, despite similar expression of GFP encoded within the same mRNA (**Fig 2B**). Despite this robust expression of Mut 4, as with Muts 1–3, there was a failure to silence *HBG1/2* mRNA and mature HbF expression in the cells, which we could show using both flow cytometry of HbF-containing cells (F cells) and by hemoglobin high-performance liquid chromatography with mature erythroid lysates (**Fig 2C–2E**). These results show that Muts 1–3 appear to either decrease the expression of BCL11A XL protein or reduce its stability, while Mut 4 has altered activity despite being well expressed in the cells.

### Examining protein stability and cellular thermal shifts of BCL11A missense variants

**Fig 3:**
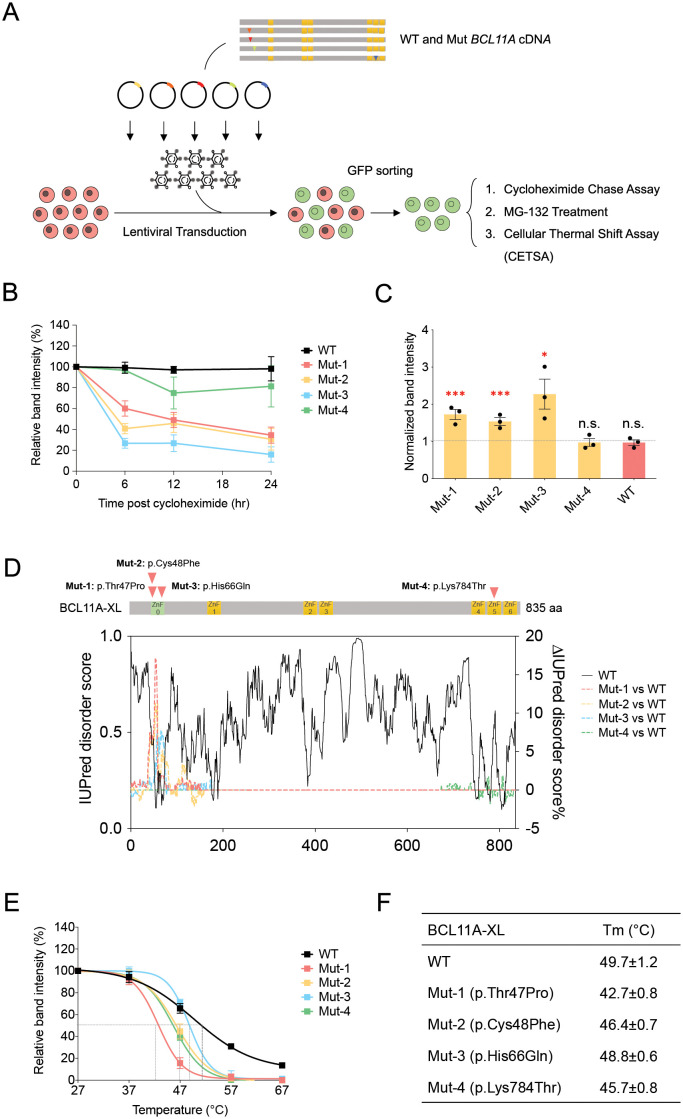
Examining protein stability and cellular thermal shifts of BCL11A missense variants. (A) Workflow of exogenous expression of either the wild type or mutant forms of the BCL11A XL in K562 cells showing lentiviral transduction, GFP sorting, protein stability and cellular thermal shifts assays. (B) Quantitative representation of BCL11A levels for Cycloheximide Chase Assay from exogenous wild type or mutant forms of the BCL11A XL cDNA expression in K562 cells. Cells were treated with 25 μg/ml cycloheximide to arrest ongoing protein synthesis and were collected for western blotting of BCL11A expression at 0hr, 6hr, 12hr and 24hr time points post cycloheximide treatment. BCL11A relative expression levels are normalized with GAPDH expression. Results are shown as mean ± SEM from three biological replicates. (C) Quantitative representation of western blotting of BCL11A levels for MG-132 treatment (10 μM, 4hr) from exogenous wild type or mutant forms of the BCL11A XL cDNA expression in K562 cells. BCL11A relative expression levels are normalized with GAPDH expression. Band intensities of all groups are relative to 0hr time point of each group. Results are shown as mean ± SEM from three biological replicates. (D) Protein disorder prediction and comparison for BCL11A mutant forms relative to wild type form using IUPred disorder score. (E) Quantitative modeling of BCL11A levels for Cellular Thermal Shift Assay from exogenous wild type or mutant forms of the BCL11A XL cDNA expression in K562 cells. Cells were treated at 27°C, 37°C, 47°C, 57°C, and 67°C for 3 minutes and were collected for western blotting of BCL11A expression. BCL11A relative expression levels are normalized with GAPDH expression. Results are shown as mean ± SEM from three biological replicates. (F) Melting temperatures (Tm) for wild type or mutant forms of the BCL11A based on modeling of BCL11A levels for Cellular Thermal Shift Assay. Results are shown as mean ± SEM from three biological replicates.

Given the reduced expression of Muts 1–3 in primary HSPC-derived cells (**Fig 2B**), we wanted to examine overall protein stability in erythroid cells. To achieve this, we used K562 erythroleukemia cells, which can be readily transduced with lentivirus in large scale for biochemical assessment, but that contain no background BCL11A protein, enabling effective monitoring of wild type or mutant proteins. We initially took sorted cells expressing the BCL11A wild type or mutant proteins and examined stability of the proteins following cycloheximide treatment of cells to arrest ongoing protein synthesis (**Figs 3A and** S2A). While the wild type and Mut 4 remained relatively stable after arresting ongoing protein synthesis over the course of 24 hours, we noted a rapid decrease in the levels of Muts 1–3 following arrest of ongoing protein synthesis (**Fig 3B**). To further delineate the basis of the observed reduced protein stability, we wondered if more rapid proteasomal degradation could underlie the rapid decrease in protein levels after arresting ongoing protein synthesis. We therefore used the reversible and cell permeable proteasome inhibitor MG-132 [34]. Upon treatment of cells expressing the wild type or mutant forms of BCL11A for 4 hours with MG-132, we noted a significant increase in the levels of Muts 1–3, while Mut 4 and the wild type protein remained at similar levels (**Fig 3C**). Crucially, treatment of primary erythroid cells with this inhibitor over 8 hours failed to alter endogenous BCL11A levels suggesting a low level of proteosomal degradation under normal conditions (**S2B Fig)**. These findings collectively show that the overall stability of these mutants in the ZnF0 domain was dramatically reduced and their degradation was promoted through the proteasome as a result [35].

We next wanted to examine cellular thermal shifts, which can enable assessment of how either a drug treatment or a mutation can alter cellular protein stability at varying temperatures [36]. Interestingly, we noted reduced cellular thermal stability for all of the mutants to varying extents (**Figs 3D, 3E, 3F, and** S2C). We would note that some of the calculations may be impacted by low expression of the mutants (e.g. Mut 3) and this may confound the Tm calculations made. It is interesting to note that Mut 4 also had reduced cellular thermal stability compared to the wild type, suggesting that specific DNA or protein co-factor binding events were being impacted as a result of this mutation, despite its comparable protein expression and stability, as the wild type (**Fig 2B**).

### Loss of DNA binding by a pathogenic missense variant in BCL11A ZnF5 underlies impaired HbF silencing

**Fig 4:**
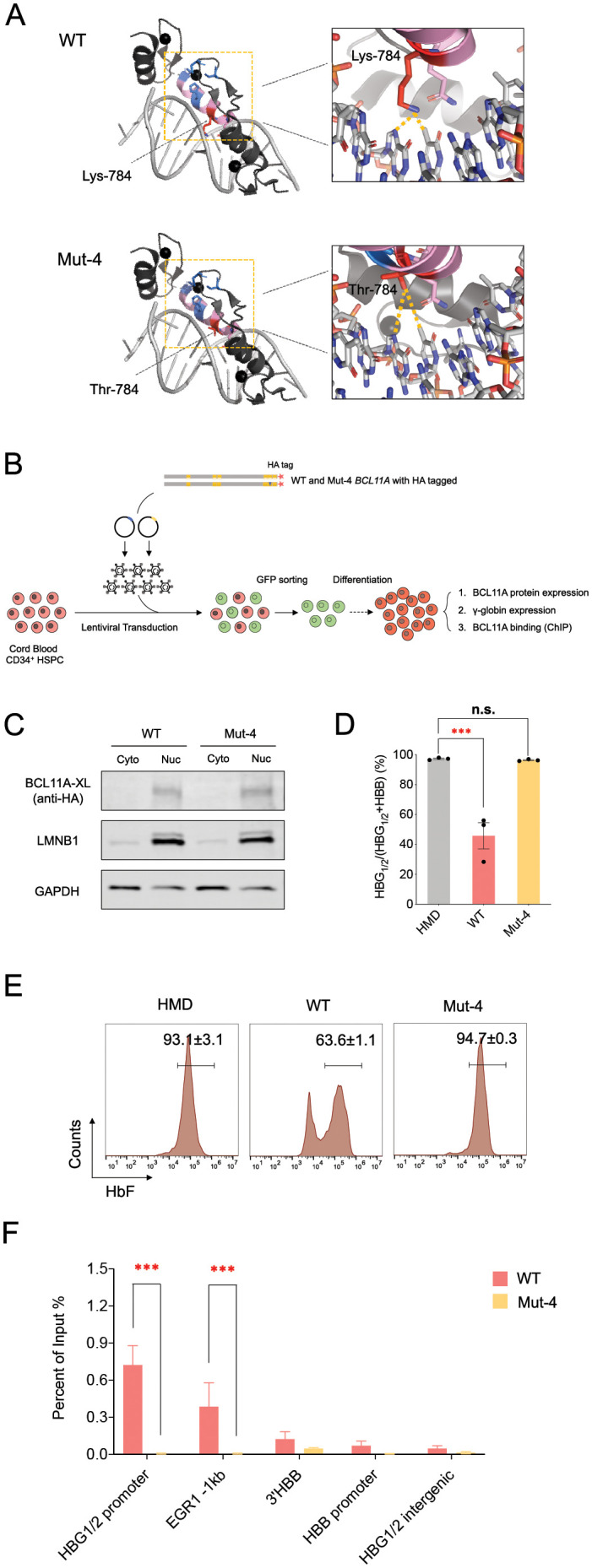
Loss of DNA binding by a pathogenic missense variant in BCL11A ZnF5 underlies impaired HbF silencing. (A) Left panel: Structure of ZnF5 for wild type and Mut 4 (p.Lys784Thr) form of BCL11A. Right panel: Zoom in view of ZnF5 indicating DNA binding alteration between wild type (Lys-784) and Mut 4 (Thr-784). (B) Workflow of exogenous expression of either the wild type or Mut 4 forms of the BCL11A XL with a HA-tag at C terminal in cord blood HSPCs showing lentiviral transduction, GFP sorting, erythroid differentiation, functional and binding analysis. (C) Western blot of BCL11A expression for nuclear/cytoplasmic fractions using antibody against HA-tag in cord blood erythroid cells expressing exogenous wild type and Mut 4 HA-tagged forms of BCL11A. LMNB1 and GAPDH as controls for nuclear fraction and cytoplasmic fraction. Representative results are shown from three biological replicates. (D) Gene expression analysis for HBG1/2 and HBB in cord blood erythroid cells expressing exogenous wild type and Mut 4 HA-tagged forms of BCL11A. Results are shown as mean ± SEM from three biological replicates (***P<0.001, n.s.: statistically non-significant by a two-tailed Student t-test). (E) Representative HbF flow cytometry on day 13 of cord blood erythroid differentiation. Results are shown as mean ± SEM from three biological replicates. (F) Chromatin binding activities of BCL11A across within the human β-globin locus in cord blood-derived erythroid cells expressing the wild type or Mut 4 HA-tagged proteins at day 11 of erythroid differentiation. Results are shown as mean ± SEM from three biological replicates (***P<0.001 by a two-tailed Student t-test).

As Mut 4 had reduced cellular thermal stability, but had similar protein expression as the wild type BCL11A, we wondered what activities may be altered by this missense variant. Based on a recent crystal structure of ZnFs 3–5 of BCL11A XL [29], we could generate a molecular model of the variant (**Fig 4A**). This model revealed the potential for altered interactions between ZnF5 and both strands of DNA upon mutation of Lys784 to Thr784. Specifically, substitution to a threonine residue ablates hydrogen bonding of the residue to G-115 and T-116 as well as to Gln781. These interactions have been reported to contribute strongly to the binding affinity for ZnF5 to DNA [29]. Therefore, Mut 4 is expected to have a reduced binding affinity for the BCL11A motif, such that key DNA binding activities would be altered. This variant would be predicted to thereby alter DNA binding by the XL isoform of BCL11A, including the critical interactions at the *HBG1/2* promoters that have been shown to have a role in HbF silencing [8,9,12].

To directly test whether this variant would result in impaired DNA binding at the *HBG1/2* promoters and other sites, we introduced a C-terminal HA tagged version of *BCL11A* wild type or Mut 4 cDNA into cord blood HSPCs that were subject to differentiation (**Fig 4B**). As with the untagged Mut 4 expression, we noted comparable erythroid differentiation (**S3A and S3B Fig**) and a failure to effectively silence HbF and *HBG1/2* mRNA expression by the mutant compared to the tagged wild type protein, despite the fact that the Mut 4 protein appropriately localized to the nucleus (**Fig 4C–4E**). We performed chromatin immunoprecipitation in cord blood-derived erythroid cells expressing the wild type or Mut 4 HA-tagged proteins at day 11 of erythroid differentiation. With this approach, we noted robust binding at well-characterized sites known to be bound by BCL11A from prior studies by the wild type, but significantly diminished binding by the Mut 4 (**Fig 4F**) [8,9,12]. This was particularly notable at the human β-globin locus on chromosome 11, where we observed reduced binding at the *HBG1/2* promoters (**Fig 4F**).

## Discussion

Here, we describe a comprehensive interrogation of all reported *BCL11A* missense variants that have been identified through studies of individuals with neurodevelopmental disorders and shown to also result in impaired HbF silencing [21]. We demonstrate how of all the reported missense variants in the context of primary human erythroid cells, the majority appear to destabilize the XL protein isoform and cause haploinsufficiency as a result, similar to the impact of deletions or other loss-of-function variants [16,17]. It is interesting to note that the destabilizing variants all impact the ZnF0 N-terminal C2HC zinc finger, which has been suggested to be dispensable for HbF silencing based on studies in mouse erythroleukemia cells [8]. This difference may indicate variation in silencing requirements between primary human cells and mouse erythroleukemia cells or, alternatively, may be attributable to protein destabilization by missense mutations that would not be the case with removal of the C2HC zinc finger completely. It is also interesting to note that these mutants in the L isoform seem to allow protein expression, but impair nuclear localization, suggesting either cell type or isoform-specific differences in how these variants perturb BCL11A activities [18]. Our findings motivate future structure-function studies in the primary human erythroid system we discuss here that may uncover key differences compared to earlier studies in transformed mouse cell lines or in other cellular contexts. Importantly, our findings also suggest a valuable approach for therapeutic targeting of BCL11A based on our functional follow up of *in vivo* observations: destabilization via this ZnF0 N-terminal C2HC zinc finger can promote proteasomal degradation and small molecules could be designed that mimic such activities and thereby enable induction of HbF levels.

We extend recent findings that suggest an important role for ZnF5 in enabling DNA binding at the *HBG1/2* proximal promoters for effective HbF silencing [8,9,12]. We demonstrate how impairment of this DNA binding event is sufficient to derepress HbF *in vivo* by using a faithful primary cell model. These findings validate the role of this critical binding event at the level of the *trans*-acting factor involved, as prior studies have only mutated specific *cis*-regulatory elements. Coupled to the structural modeling we have done, these findings suggest a critical role for DNA sequence recognition in enabling effective HbF silencing.

While additional pathogenic BCL11A variants may be discovered, four *BCL11A* variants have been identified in studies of thousands of individuals with neurodevelopmental disorders. It is possible that subtle hypomorphic mutations may be missed by ascertaining only individuals diagnosed with neurodevelopmental phenotypes. Future population-based studies that can be coupled to robust functional assays, as we illustrate here, will be valuable and enable further delineation of a possible allelic series impacting *BCL11A*. It is likely that more precise mechanistic insights may be possible through the discovery of additional *BCL11A* variants and the assays we describe could be adapted for massively parallel functional assays in the future [37].

Finally, our studies motivated by *in vivo* human observations also suggest key assays or approaches to identify potential modulators of HbF expression. For example, use of the cellular thermal shift assay for drug screening, as we have validated with pathogenic missense variants, could enable small molecules that destabilize BCL11A to be identified [36]. Even a shift in the Tm by a few degrees appeared sufficient to destabilize BCL11A to an extent that enabled high level HbF production in humans. In addition, our observation that Muts 1–3 promote proteasomal degradation of BCL11A suggests a potent approach for targeting BCL11A using small molecules. Molecular glues are being identified to promote proteasome recruitment and degradation of select proteins [38]. Such approaches could enable the development of improved therapies for HbF induction that are inspired by robust *in vivo* observations made in patients. We would note that the *in vivo* observations made in these patients also emphasize the need to ensure that such approaches target BCL11A activity selectively in the hematopoietic system and not in other impacted organs, such as the brain.

## Supporting information

S1 Fig Expression of BCL11A in cord blood CD34^+^ cells. (A) CD34^+^ cell fraction from fresh cord blood CD34 positive selection obtained from three independent healthy donors. Results are shown as mean ± SEM from three biological replicates. (B) GFP sorting of cord blood erythroid cells transduced with a co-transcribed GFP marker following an internal ribosome entry site after the *BCL11A* cDNA. Results are shown as mean ± SEM from three biological replicates. (C) Flow cytometric assessment of erythroid differentiation using CD235a and CD71 at day 10 of the differentiation process. Results are shown as mean ± SEM from three biological replicates.
(PDF)

S2 Fig Characterization of BCL11A stability. (A) GFP sorting of K562 cells transduced with a co-transcribed GFP marker following an internal ribosome entry site after the *BCL11A* cDNA. Results are shown as mean ± SEM from three biological replicates. (B) Quantitative representation of western blotting of BCL11A levels for MG-132 treatment (untreated and 5 μM for 0hr, 2hr, 4hr, 6hr, 8hr) in adult blood HSPCs at day 8 of the differentiation process. BCL11A relative expression levels are normalized with GAPDH expression. Band intensities of all time points are relative to 0hr time point. Results are shown as mean ± SEM from three biological replicates. (C) Quantitative representation of western blotting of BCL11A levels for Cellular Thermal Shift Assay from exogenous wild type or mutant forms of the BCL11A XL cDNA expression in K562 cells. Cells were treated at 27°C, 37°C, 47°C, 57°C, and 67°C for 3 minutes and were collected for western blotting of BCL11A expression. BCL11A relative expression levels are normalized with GAPDH expression. Results are shown as mean ± SEM from three biological replicates.
(PDF)

S3 Fig Characterization of BCL11A Mut-4 involving DNA binding zinc-finger domain. (A) Morphology of cord blood erythroid cells transduced with wild type or Mut 4 HA-tagged forms of BCL11A. Representative results are shown from three biological replicates. (B) Flow cytometric assessment of erythroid differentiation using CD235a, CD71 and CD49d at day 11 of the differentiation process. Results are shown as mean ± SEM from three biological replicates. (C) Western blot of BCL11A expression using antibody against HA-tag in cord blood erythroid cells expressing exogenous wild type and Mut 4 HA-tagged forms of BCL11A. Loading control is GAPDH. Representative results are shown from three biological replicates.
(PDF)

S1 Table Oligos for Site Directed Mutagenesis for BCL11A mutant constructs and qPCR primer sequences used for checking of chromatin binding events and gene expression. (XLSX)

S2 Table Western blot and Flow cytometry antibodies. (XLSX)

S3 Table Pathogenic *de novo* deletion, loss-of-function, and missense variants impacting *BCL11A* with HbF levels measured. (XLSX)
