# Supplementary figures and images for: Pathogenic BCL11A variants provide insights into the mechanisms of human fetal hemoglobin silencing
Source: PLoS Genet. 2021 Oct 11;17(10):e1009835. doi: 10.1371/journal.pgen.1009835 (PMC8530301; doi:10.1371/journal.pgen.1009835)

Fig. S1

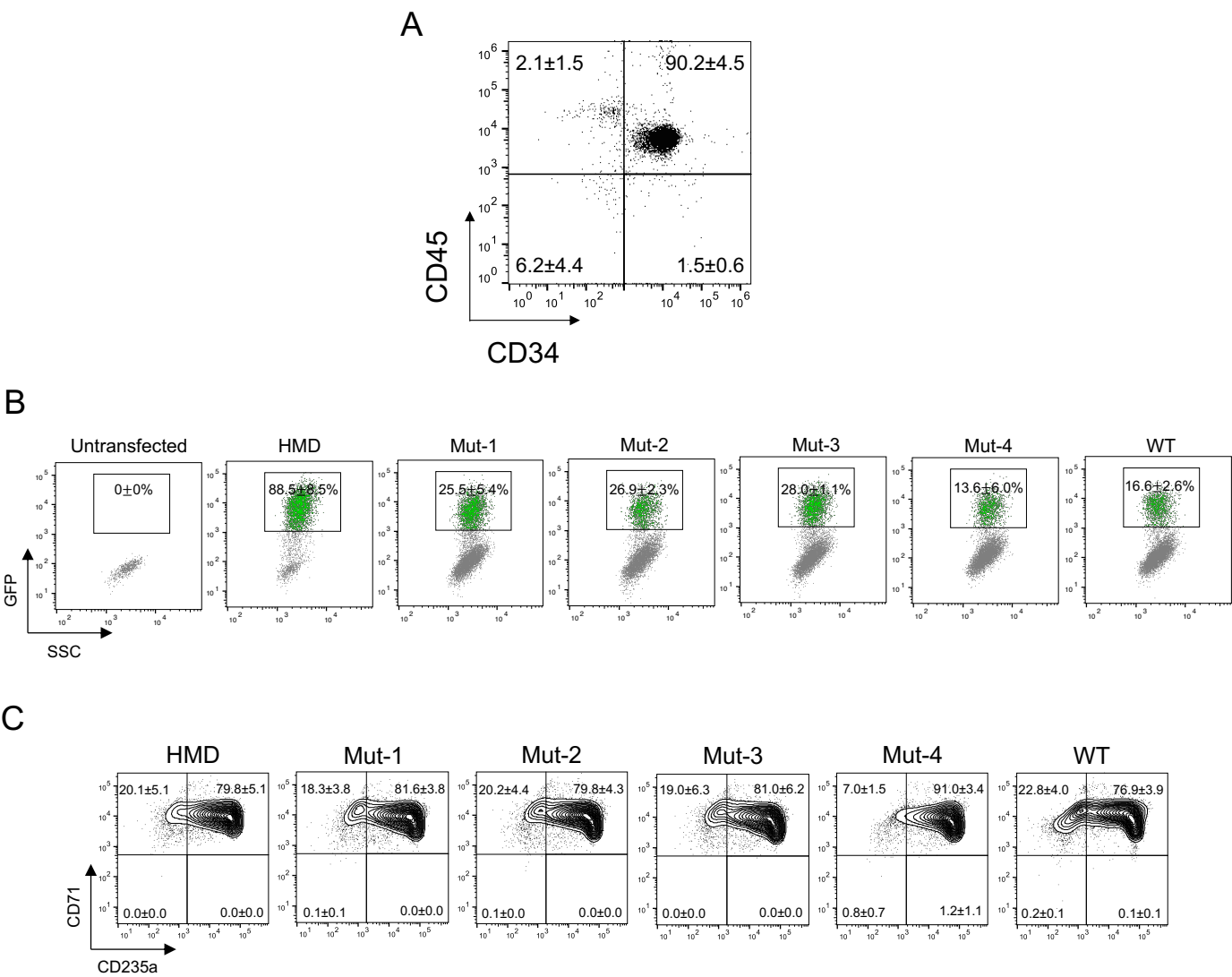

Supplement: S1 Fig — (A) CD34+ cell fraction from fresh cord blood CD34 positive selection obtained from three independent healthy donors. Results are shown as mean ± SEM from three biological replicates. (B) GFP sorting of cord blood erythroid cells transduced with a co-transcribed GFP marker following an internal ribosome entry site after the BCL11A cDNA. Results are shown as mean ± SEM from three biological replicates. (C) Flow cytometric assessment of erythroid differentiation using CD235a and CD71 at day 10 of the differentiation process. Results are shown as mean ± SEM from three biological replicates. (PDF) [file pgen.1009835.s001.pdf]

Fig. S2

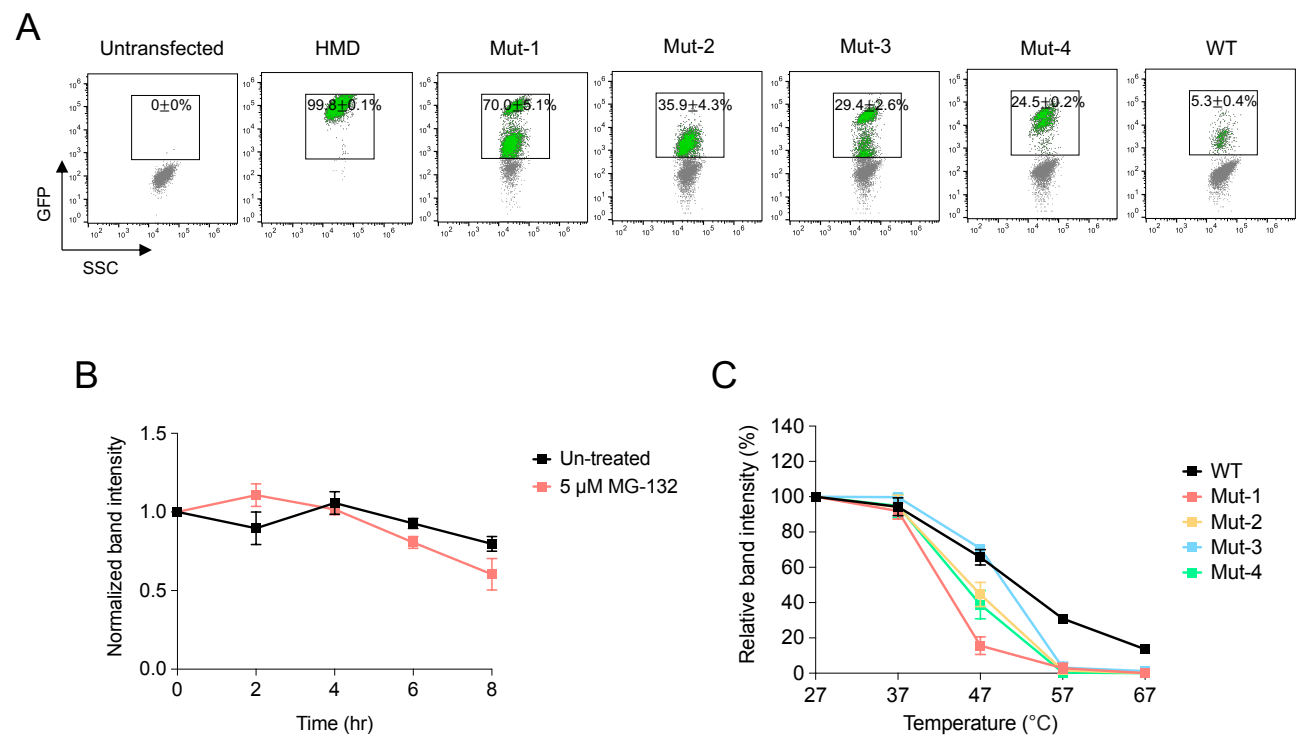

Supplement: S2 Fig — (A) GFP sorting of K562 cells transduced with a co-transcribed GFP marker following an internal ribosome entry site after the BCL11A cDNA. Results are shown as mean ± SEM from three biological replicates. (B) Quantitative representation of western blotting of BCL11A levels for MG-132 treatment (untreated and 5 μM for 0hr, 2hr, 4hr, 6hr, 8hr) in adult blood HSPCs at day 8 of the differentiation process. BCL11A relative expression levels are normalized with GAPDH expression. Band intensities of all time points are relative to 0hr time point. Results are shown as mean ± SEM from three biological replicates. (C) Quantitative representation of western blotting of BCL11A levels for Cellular Thermal Shift Assay from exogenous wild type or mutant forms of the BCL11A XL cDNA expression in K562 cells. Cells were treated at 27°C, 37°C, 47°C, 57°C, and 67°C for 3 minutes and were collected for western blotting of BCL11A expression. BCL11A relative expression levels are normalized with GAPDH expression. Results are shown as mean ± SEM from three biological replicates. (PDF) [file pgen.1009835.s002.pdf]

Fig. S3

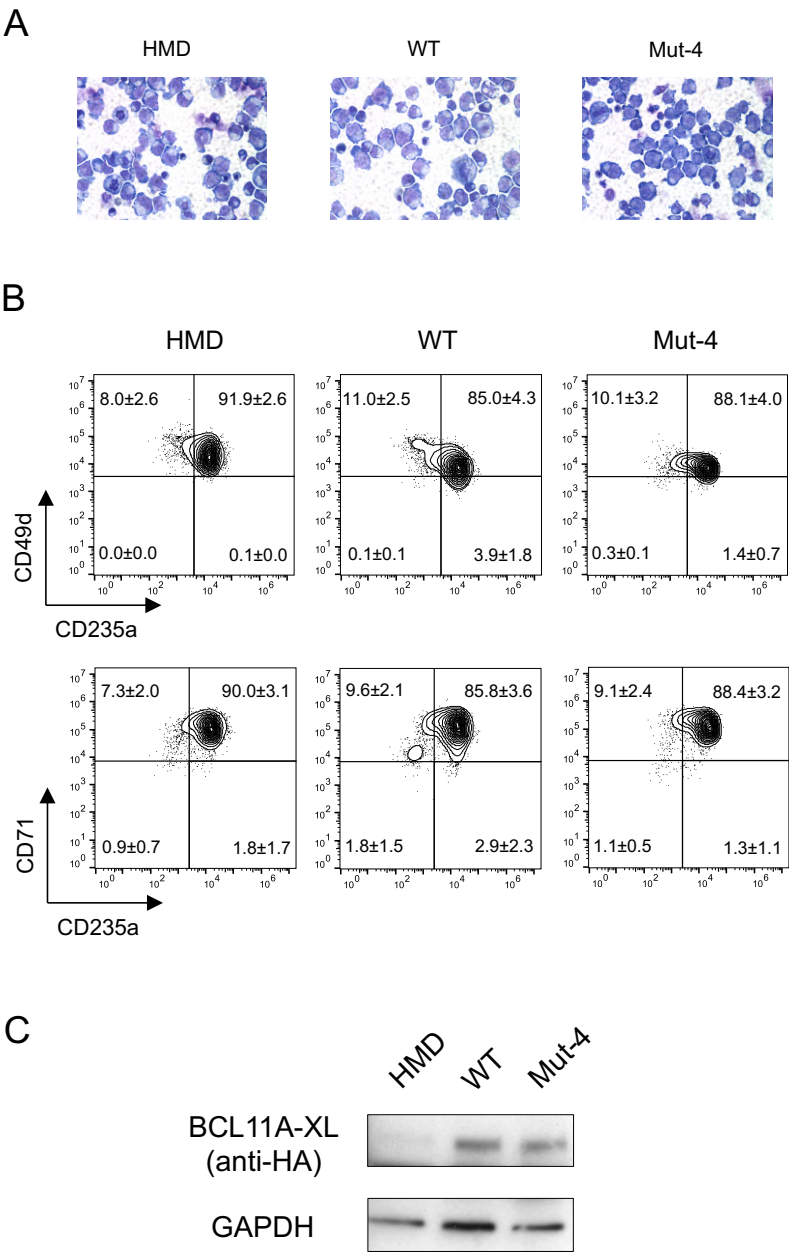

Supplement: S3 Fig — (A) Morphology of cord blood erythroid cells transduced with wild type or Mut 4 HA-tagged forms of BCL11A. Representative results are shown from three biological replicates. (B) Flow cytometric assessment of erythroid differentiation using CD235a, CD71 and CD49d at day 11 of the differentiation process. Results are shown as mean ± SEM from three biological replicates. (C) Western blot of BCL11A expression using antibody against HA-tag in cord blood erythroid cells expressing exogenous wild type and Mut 4 HA-tagged forms of BCL11A. Loading control is GAPDH. Representative results are shown from three biological replicates. (PDF) [file pgen.1009835.s003.pdf]
